# The Prognostic Value of Homocysteine in Acute Ischemic Stroke Patients: A Systematic Review and Meta-Analysis

**DOI:** 10.3389/fnsys.2020.600582

**Published:** 2021-02-12

**Authors:** Shengming Huang, Jirui Cai, Yuejun Tian

**Affiliations:** ^1^Department of Neurology, Luohe Central Hospital, The First Affiliated Hospital of Luohe Medical College, Luohe, China; ^2^Department of Cardiology, Luohe Central Hospital, The First Affiliated Hospital of Luohe Medical College, Luohe, China; ^3^Institute of Urology, Lanzhou University Second Hospital, Lanzhou University, Lanzhou, China

**Keywords:** homocysteine, acute ischaemic stroke, outcome, meta-analysis, systematic review

## Abstract

**Background:** This comprehensive meta-analysis aimed to assess whether an increased homocysteine (Hcy) level is an independent predictor of unfavorable outcomes in acute ischemic stroke (AIS) patients.

**Methods:** A comprehensive literature search was conducted up to August 1, 2020 to collect studies reporting Hcy levels in AIS patients. We analyzed all the data using Review Manager 5.3 software.

**Results:** Seventeen studies with 15,636 AIS patients were selected for evaluation. A higher Hcy level was associated with a poorer survival outcome (OR 1.43, 95% CI: 1.25–1.63). Compared with the AIS group, Hcy levels were significantly lower in the healthy control patients, with an SMD of 5.11 and 95% CI (1.87–8.35). Analysis of the different subgroups of AIS demonstrated significant associations between high Hcy levels and survival outcomes only in Caucasian and Asian patients. Moreover, whereas high Hcy levels were closely associated with gender, B12 deficiency, smoking, and patients who received tissue plasminogen activator treatment, no significant difference was found between increased Hcy levels and age, drinking, hypertension, diabetes mellitus, and hyperlipidemia. In addition, the cut-off value (20.0 μmol/L) might be an optimum cut-off index for AIS patients in clinical practice.

**Conclusion:** This meta-analysis reveals that the Hcy level may serve as an independent predictor for unfavorable survival outcomes in AIS patients, particularly in Caucasian and Asian AIS patients. Further studies can be conducted to clarify this relationship.

## Introduction

Acute ischemic stroke (AIS) occurs primarily because of atherothrombotic or embolic vascular obstruction in vessels supplying blood to the brain, leading to the death of brain tissue and focal neurological deficits and is a serious public health concern (Smith et al., 2019; Jensen and Thomalla, 2020; Phipps and Cronin, 2020). It is the main cause of physical disability and the second leading cause of death worldwide, especially in developing countries (Feigin et al., 2015; Virani et al., 2020). Therefore, the identification of modifiable risk factors for the early screening, prognostic evaluation, and intensive treatment of AIS are in active demand.

In the past year, numerous epidemiological studies have supported that homocysteine (Hcy) is a non-traditional risk factor for ischemic stroke (Homocysteine Studies, 2002; Schwammenthal and Tanne, 2004). Elevated Hcy levels at AIS onset might also be related to increased hematoma volume, and increased Hcy levels may cause endothelial dysfunction, neurotoxicity, and the upregulation of thrombosis formation factors (Luo et al., 2019). For the past decade, numerous studies have also confirmed that elevated Hcy levels are associated with functional disability for AIS and recurrent strokes (He et al., 2014). A multicenter study performed by Kwon et al. revealed that high levels of serum HCY were independent predictors of early neurological deterioration in AIS patients (Kwon et al., 2014).

Nevertheless, it is unknown whether Hcy is an independent risk parameter for worse clinical outcome in AIS patients. Although the high expression of Hcy in AIS patients has been reported, its effectiveness in prognosis prediction is still under debate. Some results confirmed that Hcy in fact correlated with worse outcomes of AIS (Ling et al., 2018; Luo et al., 2019), whereas some other studies indicated that elevated Hcy levels are not significantly associated with or an independent predictor of AIS patients' clinical outcome (Bushnell and Goldstein, 2002; Ribo et al., 2004; Wang et al., 2017).

The best method to judge and evaluate Hcy in clinical practice remains under debate. Furthermore, the roles of Hcy levels in AIS patients' clinical outcomes have not been thoroughly explored. In this work, we aim to conduct a systematic evaluation of the prognostic value of Hcy levels in relation to AIS risk.

## Materials and Methods

### Literature Search and Selection Criteria

A comprehensive search was conducted in PubMed, Cochrane, Embase, and Web of Science for relevant articles published up to August 1, 2020. The search terms were (Hcy OR homocysteine OR tHcy OR total homocysteine OR hyperhomocysteimia OR HHcy) AND (“Acute ischaemic stroke” OR AIS OR ischaemic stroke OR IS OR stroke) and “outcome or clinical outcome or survival outcome or prognosis or prognostic.”

The following criteria were used to select eligible studies: (1) Hcy level evaluated in AIS patients, (2) assessment of the correlation between Hcy level and the pathogenesis of AIS, (3) sufficient and effective information to extract the OR of outcome and 95% CIs, and (4) published in English with peer review. We excluded the following studies: (1) duplicate data presented at conferences and in case reports; (2) reviews, invalid data, or abstracts only; (3) studies of animal models; and (4) studies with insufficient data to obtain ORs and no clear Hcy results. For duplicate publications, only the most recent comprehensive results with the highest-quality size were selected.

### Data Extraction

We extracted data on the study population (the first author's name, publication date, and country) and sample parameters [number of cases, median or mean of patient age, sex, tissue plasminogen activator (tPA) treatment, smoking, drinking, hypertension, diabetes mellitus, hyperlipidemia, cut-off value of Hcy levels]. If the results of multivariate analysis and univariate analysis were both provided, the former was selected. All data were obtained by two independent observers (SMH and YJT). Study quality was assessed according to the NOS criteria for retrospective studies (Stang, 2010). If data could not be extracted from the article, we considered the relevant data not available.

### Statistical Analysis

Review Manager 5.3 (the Cochrane Collaboration) and STATA 16.0 (Stata Corporation) were used for the analysis. The pooled OR and its corresponding 95% CI were determined to assess the correlation between Hcy level and AIS patient outcome. ORs and 95% CIs were used to evaluate the relationships between Hcy level and clinical characteristics, including sex, smoking, drinking, tPA, hypertension, diabetes mellitus, and hyperlipidemia. Heterogeneity among these studies was assessed using the *I*^2^ statistic (Zintzaras and Ioannidis, 2005). A fixed-effects model (Mantel–Haenszel method) was used to calculate pooled ORs when there was no significant heterogeneity among studies (*I*^2^ > 50% suggested high heterogeneity); otherwise, the random-effects model was used. A *p*-value < 0.05 was considered to indicate significance. Publication bias was assessed using funnel plots and Egger's test (Peters et al., 2006).

## Results

### Eligible Studies and Quality Assessment

**Figure 1:**
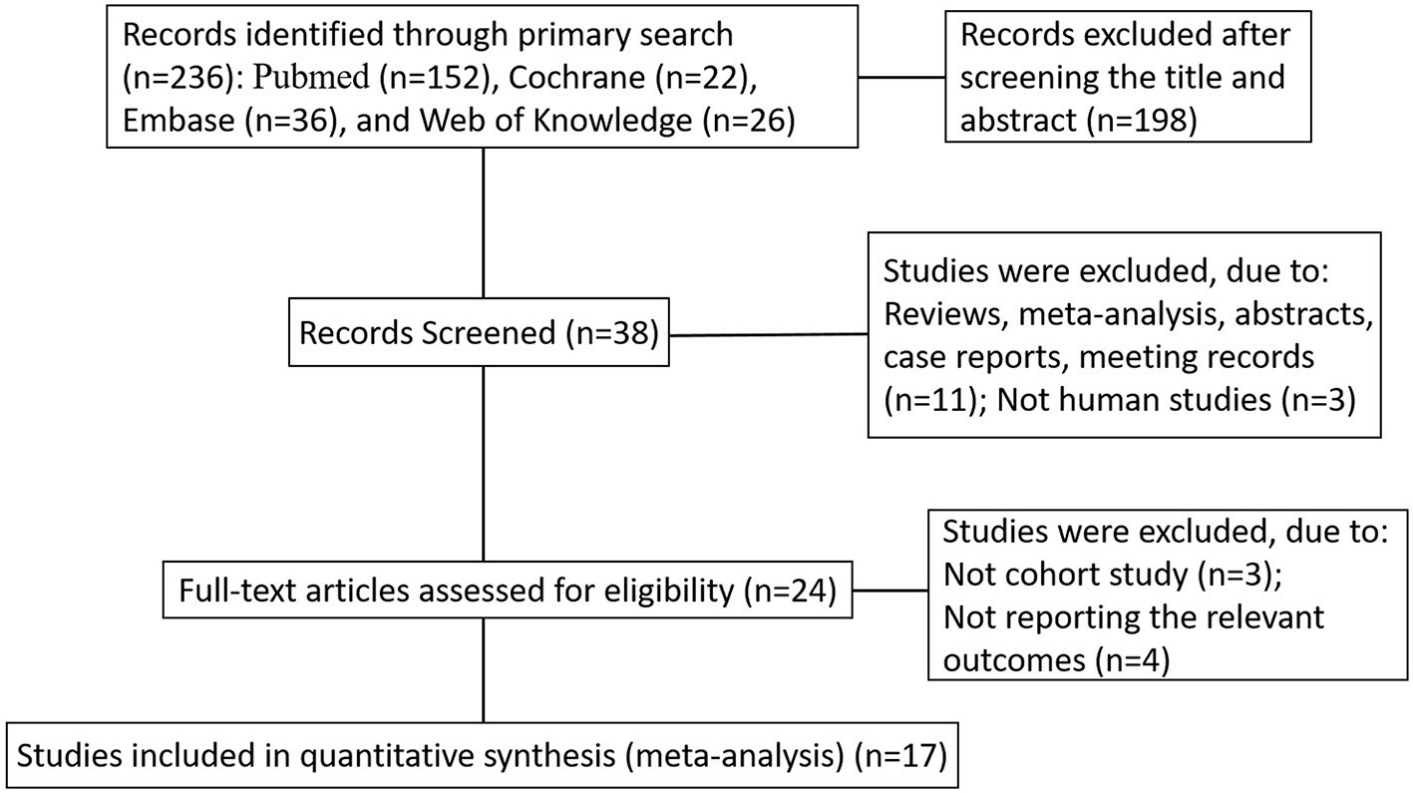
Flow chart shows study selection procedure.

The initial search identified 236 articles. Eventually, 17 articles published from 2001 to 2020 were included (Fallon et al., 2001; Sacco et al., 2004; Mizrahi et al., 2005; Perini et al., 2005; Yoldas et al., 2007; Okubadejo et al., 2008; Tu et al., 2013; Unal et al., 2013; Kwon et al., 2014; Shi et al., 2015; Forti et al., 2016; Tang et al., 2016; Yao et al., 2016; Markaki et al., 2017; Zhong et al., 2017; Alfieri et al., 2020; Li L. et al., 2020) (Figure 1). All studies reported follow-up time. Four of them were retrospective studies, and the remaining 13 were prospective studies. One prospective cohort included only men (Fallon et al., 2001), and one retrospective study cohort included only women (Markaki et al., 2017).

Of the 17 studies, six were conducted in China, two in Italy, two in Turkey, one in South Africa, one in Israel, one in the USA, one in Sweden, one in Brazil, one in Nigeria, and one in South Korea. The characteristics of these 17 studies and of a study of high methodological quality according to guidelines developed by the Newcastle–Ottawa Scale are shown in Table 1. Differences in the cut-off value for Hcy level were observed among the studies.

**Table 1:** Summary of the characteristics of enrolled studies.

**Study (year)**	**Country**	**Design**	**Number (case/control)**	**Age (mean ± SD), years**	**Male (%)**	**Cut-off (μmol/L)**	**Follow-up**	**NOSscores**
Fallon et al. (2001)	South Africa	Prospectivecohort study	2,254	50–64	0	<8.2 vs. ≥19.0	10.2 months	8
Sacco et al. (2004)	USA	Prospective study	2,939	69 ± 10	1793 (61%)	Q1, <10 vs. ≥10; Q1, <15 vs. ≥15	5 years	9
Mizrahi et al. (2005)	Israel	Retrospective study	113	72.5 ± 10.6	77 (68.1%)	≤ 15 vs. >15	18 months	8
Perini et al. (2005)	Italy	Prospective study	775/421	71.6 ± 12.1	399 (51.5%)	≤ 18.6 vs. >18.6	14 days	7
Yoldas et al. (2007)	Turkey	Prospective study	40/40	69 ± 11	NA	NA	10 days	6
Okubadejo et al. (2008)	Nigeria	Prospective study	69/86	58.8 ± 9.8	38 (55.1%)	NA	4 weeks	7
Tu et al. (2013)	China	Prospective study	189	66 (IQR, 58–75)	117 (61.9%)	≤ 15 vs. >15	3 months	7
Unal et al. (2013)	Turkey	Prospective study	41/33	74 ± 9.6	17 (41.5%)	NA	3 months	7
Kwon et al. (2014)	South Korea	Prospective study	396	63.5	240 (60.6%)	Q1, 4.67–8.38; Q2, 8.39–10.30; Q3, 10.30–13.10; Q4, 13.10–42.76	7 days	8
Shi et al. (2015)	China	Prospective study	3799	62 (IQR 54–71)	2872 (75.6%)	Q1, ≤10; Q2, 10–12.9; Q3, 12.9–18.6; Q4, >18.6	48 months	8
Tang et al. (2016)	China	Prospective study	226	NA	124 (54.9%)	≥15.5 vs. <15.5	6 months	8
Yao et al. (2016)	China	Retrospective study	194	62.2 ± 12.3	118 (60.8%)	Q1, 2.26–11.5; Q2, 11.5–15.5; Q3, 15.5–23.85; Q4, 23.85–138.90	3 months	8
Forti et al. (2016)	Italy	Prospectivestudy	644	80.3 ± 8.7	314 (48.7%)	Q1, <16; Q2, 16–29.9; Q3 ≥30	48 days	7
Zhong et al. (2017)	China	Prospective study	3309	62.4 ± 10.9	2115 (63.9%)	≤ 15 vs. >15	3 months	8
Markaki et al. (2017)	Sweden	Retrospective study	331	NA	196 (59.2%)	<13.0 vs. ≥13.0	10 years	8
Li L. et al. (2020)	China	Retrospective study	141	IQR, 58–76	93 (65.9%)	NA	3 months	8
Alfieri et al. (2020)	Brazil	Prospective cross-sectional study	176/176	67.7 ± 12.1	103 (58.52)	NA	3 months	8

*AIS, acute ischemic stroke; NA, not available; IQR, interquartile range; NOS, Newcastle–Ottawa Scale*.

### Correlation of High Hcy Levels With Survival Outcomes in AIS Patients

Of the 15 studies that evaluated the association between Hcy level and AIS survival outcome, six involved Caucasian patients (*n* = 4,981), seven involved Asian patients (*n* = 8254), and two involved African patients (*n* = 2,323).

**Figure 2:**
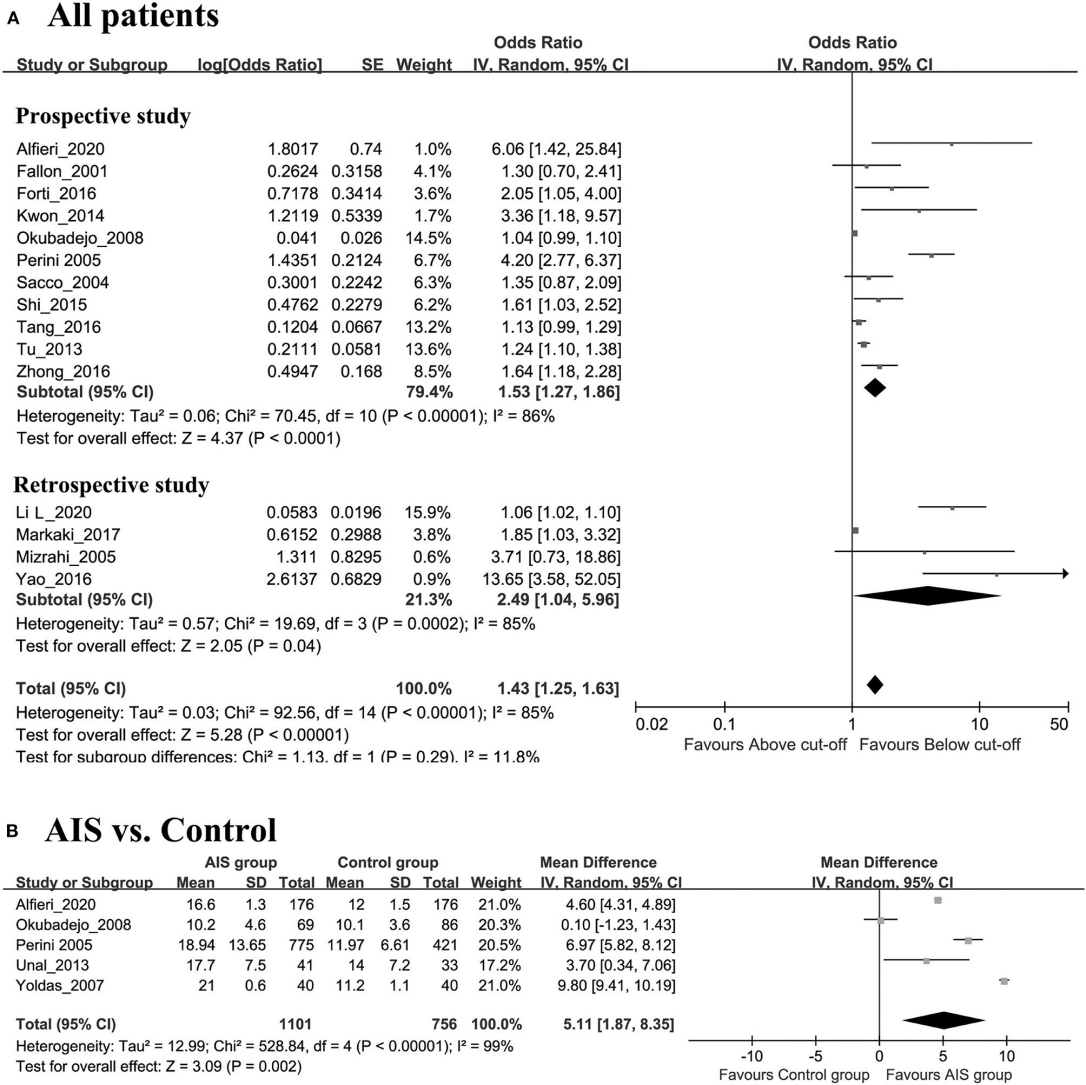
**(A)** Forest plot of study-specific relative risk statistics for AIS comparing the highest and lowest Hcy category group. **(B)** Forest plots of the subgroup analyses on sample size in relation to Hcy levels between AIS patients and healthy controls.

The comprehensive OR for 13 prospective studies was 1.53 (95% CI 1.27–1.86, *p* < 0.0001, *I*^2^ = 85%, Figure 2A). The comprehensive OR for four retrospective studies was 2.49 (95% CI 1.04–5.96, *p* < 0.0001, *I*^2^ = 86%, Figure 2A). The comprehensive OR for all AIS patient outcomes was 1.43 (95% CI 1.25–1.63, *p* < 0.0001, *I*^2^ = 89%, Figure 2A and Table 2).

**Table 2:** Results of subgroup analysis of the association between Hcy level and outcome of AIS.

**Outcome**	**Studies (n)**	**Patients**	**OR**	**95% CI**	***P*-value**	**Model**	**Heterogeneity**
							**χ^2^, I^2^, *p*-value**
All study	15	15,555	1.43	1.25–1.63	0.00	Random	92.56, 85%, 0.00
Prospective study	11	14,776	1.53	1.27–1.86	0.00	Random	70.45, 86%, 0.00
Retrospective study	4	779	2.49	1.04–5.96	0.00	Random	19.69, 85%, 0.0006
Caucasian	6	4978	2.46	1.49–4.05	0.0004	Random	16.33, 69%, 0.29
Asian	7	8254	1.22	1.08–1.38	0.001	Random	30.78, 81%, 0.00
African	2	2323	1.04	0.99–1.10	0.10	Fixed	0.49, 0%, 0.48

*I^2^, I-squared; OR, odds ratio; Fixed, fixed, inverse variance model; Random, random, I-V heterogeneity model; tPA, tissue plasminogen activator*.

**Figure 3:**
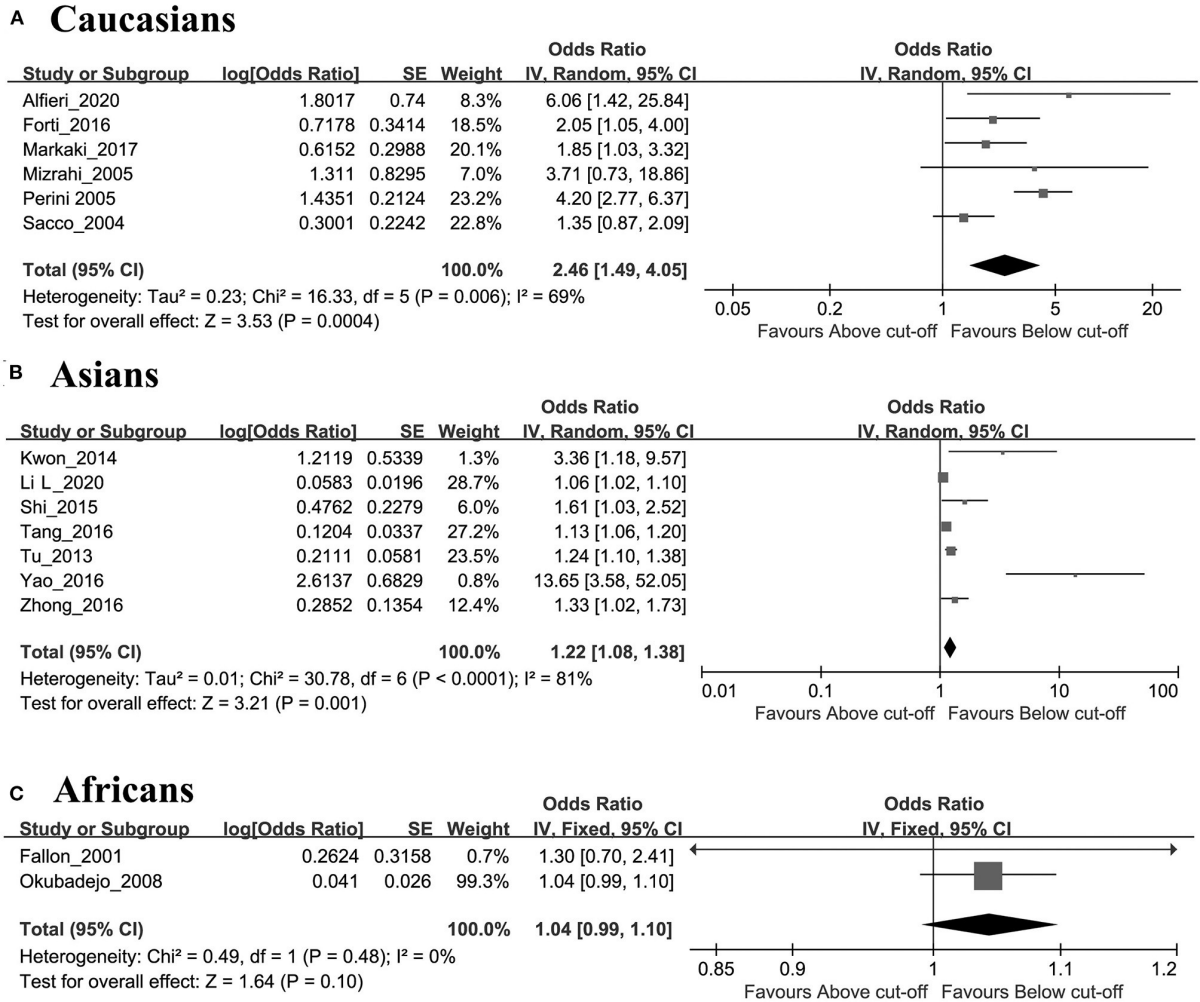
Forest plots of the subgroup analyses on ethnicity in relation to Hcy levels and risk of AIS patients: **(A)** Caucasians, **(B)** Asians, **(C)** Africans.

As shown in Figure 2B, the AIS group had significantly higher levels of Hcy than the control group (SMD = 5.11, 95% CI = 1.87–8.35, *p* = 0.002). The results of subgroup analyses on ethnicity showed that the associations were statistically significant in both Caucasian and Asian patients (Caucasian: OR 3.56, 95% CI 2.54–4.98, *p* < 0.00001, *I*^2^ = 20%, Figure 3A; Asian: HR 1.39, 95% CI 1.19–1.63, *p* < 0.00001, *I*^2^ = 86%, respectively, Figure 3B) but not in African patients (OR 1.04, 95% CI 0.99–1.10, *p* = 0.11) (Figure 3C).

### Relationships Between Hcy Level and Clinical Parameters in AIS Patients

Five studies investigated the association of clinical characteristics such as sex and hypertension with elevated Hcy levels. Four studies investigated the relationship between smoking and Hcy levels. Three studies assessed the relationship between age and Hcy levels. Two studies assessed the association of clinical characteristics such as drinking, tPA, diabetes mellitus, and hyperlipidemia with elevated Hcy levels. One study assessed the relationship between B12 deficiency and Hcy levels (Table 3).

**Table 3:** Results of subgroup analysis of the association between Hcy level and clinic parameters in AIS.

**Outcome of interest**	**Studies (n)**	**Patients**	**OR/SMD**	**95% CI**	***P*-value**	**Model**	**Heterogeneity**
							**χ^2^, I^2^, *p*-value**
AIS vs. control	5	1,857	SMD: 5.11	1.87–8.35	0.002	Random	528.84, 99%, 0.00
Age	3	642	SMD: 1.54	−1.36 to 4.45	0.30	Random	5.05, 60%, 0.08
Sex	5	1,512	OR: 1.14	1.06–1.23	0.0007	Fixed	36.92, 89%, 0.00
Smoking	4	4,720	OR: 0.66	0.57–0.75	0.00	Fixed	5.32, 44%, 0.15
Drinking	2	509	OR:0.65	0.30–1.41	0.28	Random	3.87, 74%, 0.28
tPA	2	335	OR: 1.06	1.02–1.10	0.003	Fixed	1.33, 25%, 0.25
Hypertension	5	4,833	OR: 0.78	0.55–1.10	0.15	Random	12.19, 67%, 0.02
Diabetes mellitus	2	509	OR: 0.89	0.37–2.14	0.79	Random	3.13, 68%, 0.08
Hyperlipidemia	2	509	OR: 1.04	0.71–1.52	0.84	Fixed	0.00, 0%, 0.97
B12 deficiency	1	2,939	SMD: 5.00	4.23–5.77	0.00	Fixed	NA

*I^2^, I-squared; Fixed, fixed, inverse variance model; Random, random, OR, odds ratio; tPA, tissue plasminogen activator; I-V heterogeneity model; SMD, standardized mean difference*.

There were statistically significant differences between Hcy levels in relation to sex, smoking, tPA, and B12 deficiency. The pooled ORs were as follows: OR 1.14, *p* = 0.0007; OR 0.66, *p* < 0.001; OR 1.06, *p* = 0.003; and SMD 5.0, *p* < 0.001, respectively. There were no significant differences in relation to the Hcy level and age, drinking, hypertension, diabetes mellitus, or hyperlipidemia in AIS patients. The pooled ORs were as follows: (SMD 1.54, *p* = 0.30; OR 0.65, *p* = 0.28; OR 0.78, *p* = 0.15; OR 0.89, *p* = 0.79; and OR 1.04, *p* = 0.84, respectively) (Table 3).

### Relationships Between High Hcy Levels and Survival Outcomes in AIS Patients Using Different Cut-Off Values

Analysis data for all cut-offs (including 13.0, 16.5, and 20.0 μmol/L) were used to elucidate the optimal cut-off of the Hcy level. The combined ORs and 95% CIs were as follows: 1.33 (95% CI 0.86–2.07) vs. 1.71 (95% CI 1.36–2.17) for a cut-off value of 13.0 μmol/L (Supplementary Figure 1), 1.29 (95% CI 1.13–1.49) vs. 2.62 (95% CI 0.74–9.33) for a cut-off value of 16.5 μmol/L (Supplementary Figure 2), and 1.96 (95% CI 1.39–2.75) vs. 1.59 (95% CI 1.16–2.19) for a cut-off value of 20.0 μmol/L (Supplementary Figure 3). In terms of both low and high cut-off values to assess high Hcy reactivity, subgroup analysis uncovered a significant correlation of high Hcy reactivity with worse survival outcome (*p* < 0.05, Supplementary Table 1).

The cut-off value (20.0 μmol/L) resulted in a significantly higher association with outcome than other values, indicating that the cut-off value (20.0 μmol/L) might be a superior cut-off index for clinical practice.

### Publication Bias

**Figure 4:**
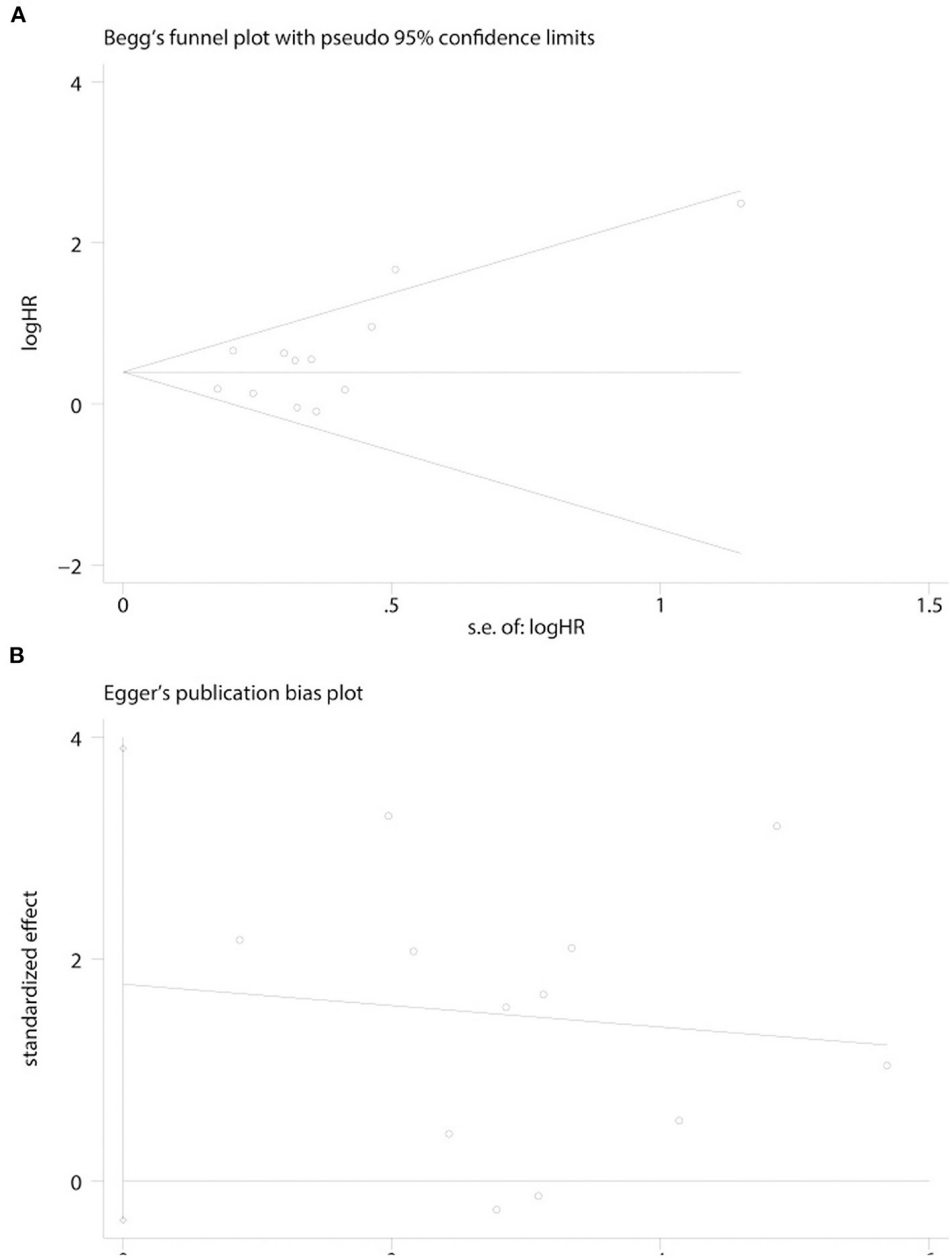
Funnel plots were used to evaluate publication bias on survival outcome. **(A)** Begg's test was not significant, intending no significant bias was observed on survival outcome. **(B)** Egger's test was not significant, intending no significant bias was observed on survival outcome.

Publication bias was calculated by Begg's test and Egger's test for the outcome of AIS. Begg's test (*p* = 0.244) and Egger's test (*p* = 0.171) suggested that our results were stable (Figure 4).

## Discussion

Hcy is a sulfur-containing amino acid that is widely known to be involved in the pathogenesis of multiple clinical conditions. Elevated Hcy levels have been shown to be related to cardiovascular disease, Alzheimer dementia, and cerebrovascular diseases, suggesting endothelial dysfunction as a major mechanism (Hooshmand et al., 2013; Chrysant and Chrysant, 2018). Moreover, reports have demonstrated the increased risk of stroke in individuals homozygous for the MTHFR T allele is close to the result predicted by the differences in homocysteine concentration given by this variant (Casas et al., 2005). In addition, the elevated level of Hcy promotes neural stem cell autophagy in both *in vivo* and *in vitro* models of AIS (Wang et al., 2019). However, the relationship between Hcy and prognosis remains unclear, and the roles and clinical implications of Hcy levels in AIS have not been thoroughly studied.

In our meta-analysis, the pooled data indicated the following: (1) AIS patients with a higher Hcy level had a worse survival outcome, and the Hcy level was higher in AIS patients than in healthy controls; (2) a high Hcy level was associated with sex and smoking and was not strongly associated with drinking, hypertension, diabetes mellitus, age, and hyperlipidemia in AIS patients; and (3) the cut-off value of 20.0 μmol/L might be superior to other cut-off values for clinical practice. (4) Hcy may serve as an independent risk factor influencing the survival outcome of AIS patients, particularly in Caucasians and Asians. Furthermore, our findings highlight that the Hcy level is a prognostic factor for AIS and suggest that thrombolytic therapy may be helpful in the high-risk subgroup of patients (Yao et al., 2016; Li L. et al., 2020).

In addition, we found a significant association between Hcy levels and vitamin B12 deficiency. In a subgroup analysis among 2,939 subjects, the effect of tHcy remained significant on conventional serum vitamin B12 levels. Several studies have shown that daily vitamin B supplementation has a significant protective effect on recent stroke or transient ischemic attack (Huang et al., 2012). One issue that needs to be addressed is to identify the impact of modifiable risk factors (i.e., hyperhomocysteinemia) on clinical practice, such as suggesting homocysteine-lowering interventions, like supplementation with B vitamins, to improve the prognosis of AIS patients or to reduce the risk of stroke in primary prevention in the high-risk subgroup of patients.

The biological role of Hcy explains its survival outcome and clinical role in AIS. Elevated Hcy levels suggest severe neuropathy and an increased volume of hemorrhage (Shandal and Luo, 2016; Zhou et al., 2018) because elevated plasma homocysteine can cause injury to vascular wall integrity and cerebrovascular permeability, which may result in endothelial dysfunction, elastic structure damage, and vascular endothelial damage and further damage the basic layer of cerebral arterioles and microvessels (Fan et al., 2017). Hyperhomocysteinemia has been widely regarded as an important risk factor in the formation of atherosclerosis, indicating that elevated Hcy levels are closely related to endothelial dysfunction (Lu et al., 2018; Borowczyk et al., 2019). Furthermore, high levels of Hcy also increase LDL oxidation, and the main mechanism of Hcy adverse effects on vascular endothelial function involves oxidative stress and the consumption of bioactive nitric oxide (Seo et al., 2010; AnandBabu et al., 2019).

Our results confirmed that high Hcy level was related to the gender of AIS patients. Moreover, gender has been shown to have an effect on the level of Hcy (Li J. et al., 2020), and recent studies have shown that there are significant gender differences in the relationship between Hcy and the risk of ischemic stroke disease (Zhong et al., 2017; Foscolou et al., 2019). Therefore, there may be a sex-specific correlation between Hcy and functional disability or cardiovascular events in patients with AIS.

The data demonstrated that the cut-off point of 20 μmol/L was superior to the cut-off points of 13.0 and 16.5 μmol/L in AIS patients. Several epidemiological studies have indicated that a wide range of cut-off points for Hcy is a modifiable risk factor. The present results indicated that the elevated Hcy levels to 18.6 μmol/L had a 1.61-fold increased risk of death compared with patients in the lowest quartile (10 μmol/L) (Shi et al., 2015). Based on the meta-analysis, we judged that the optimal cut-off value of Hcy was 20.0 μmol/L, and a similar cut-off point for Hcy (23.85 μmol/L) was shown by Yao et al. (2016). Thus, a cut-off point of 20.0 μmol/L Hcy is the most effective risk factor for AIS patients.

In addition, subgroup analysis by ethnicity revealed that in both Caucasians and Asians, the level of serum Hcy was significantly related to the survival time of AIS patients but not in African ASI patients. The inconsistency may be related to factors such as environment, lifestyles, and living conditions.

Our study also has some limitations. First, the employed methods for detecting serum Hcy levels and cut-off values differed, which may result in sensitivity and reliability issues. Second, clinical factors including blood pressure, blood glucose, smoking, drinking, diabetes mellitus, hypertension, and hyperlipidemia in each study may lead to bias. These variances may have produced heterogeneity in this study. Third, there was a small study impact due to the limited sample size of our study. There is thus certainly a need for large-scale trials to investigate the roles of other factors that are likely to cause AIS.

This meta-analysis is the first study to systematically assess the correlation of the Hcy level with clinical characteristics and its prognostic role in AIS. Elevated Hcy levels may contribute to disease progression and are a significant risk factor affecting survival outcome, and the reliable convenience and low cost of Hcy measurement should further facilitate its wider application in patients with AIS. The evaluation of the Hcy level should raise the possibility of employing an aggressive treatment strategy.

## Data Availability Statement

The original contributions presented in the study are included in the article/Supplementary Material, further inquiries can be directed to the corresponding author.

## Author Contributions

SH, JC, and YT wrote the main manuscript text and approved it. All authors contributed to the article and approved the submitted version.

## Conflict of Interest

The authors declare that the research was conducted in the absence of any commercial or financial relationships that could be construed as a potential conflict of interest.
