# Supplementary figures and images for: The Prognostic Value of Homocysteine in Acute Ischemic Stroke Patients: A Systematic Review and Meta-Analysis
Source: Front Syst Neurosci. 2021 Feb 12;14:600582. doi: 10.3389/fnsys.2020.600582 (PMC7907516; doi:10.3389/fnsys.2020.600582)

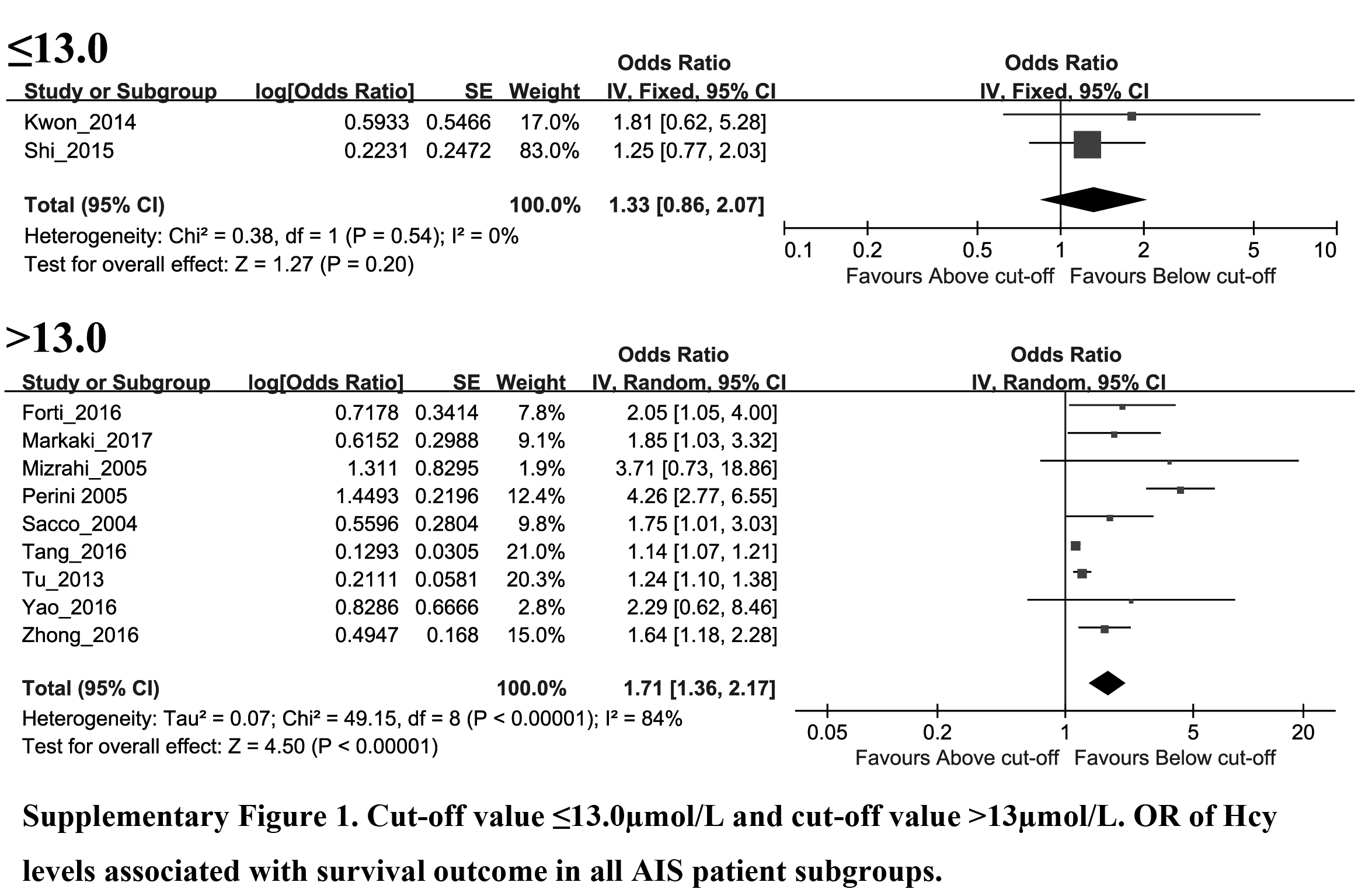

Supplement: Supplementary file 1 [file Image_1.tif]

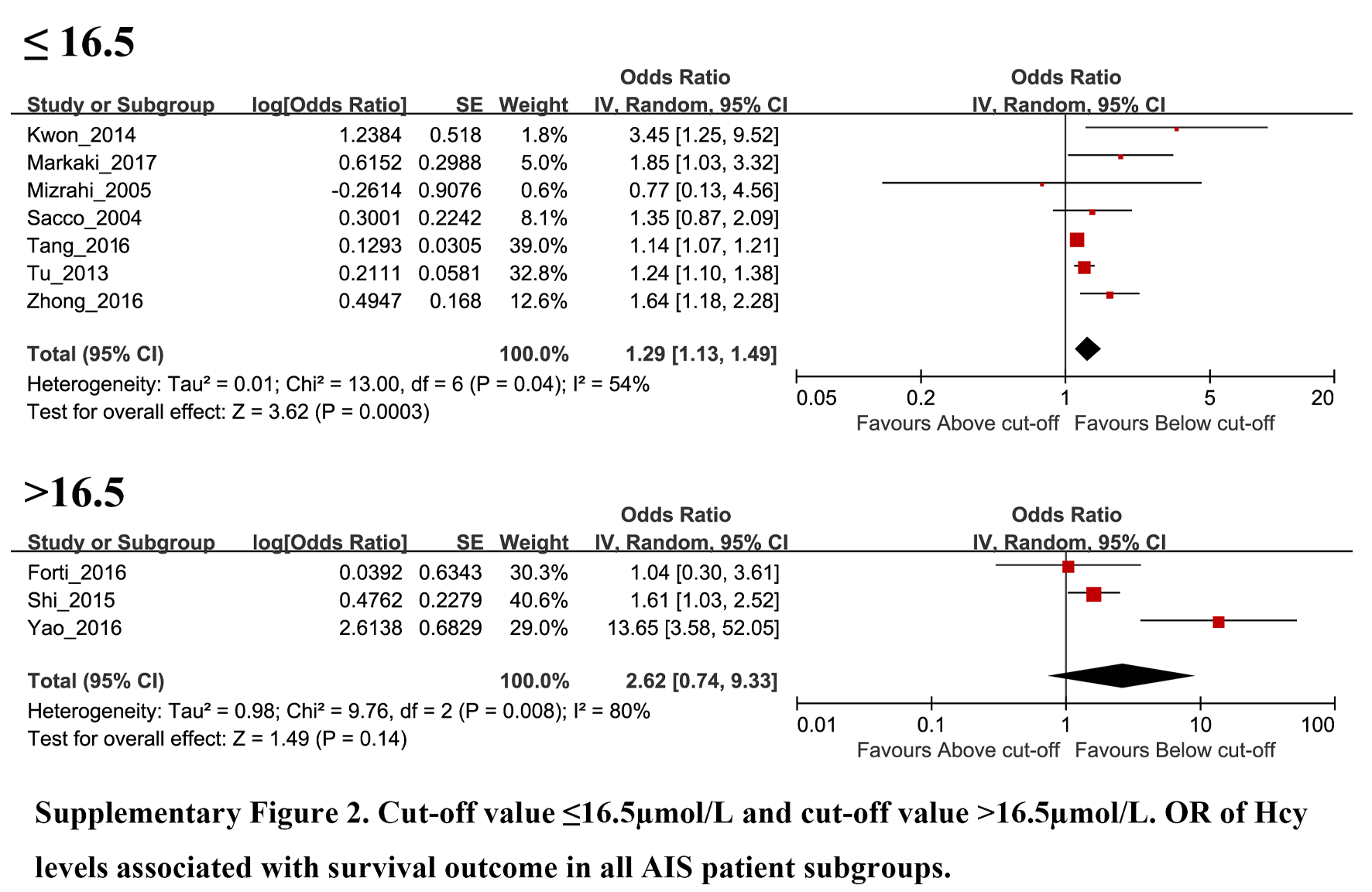

Supplement: Supplementary file 2 [file Image_2.tif]

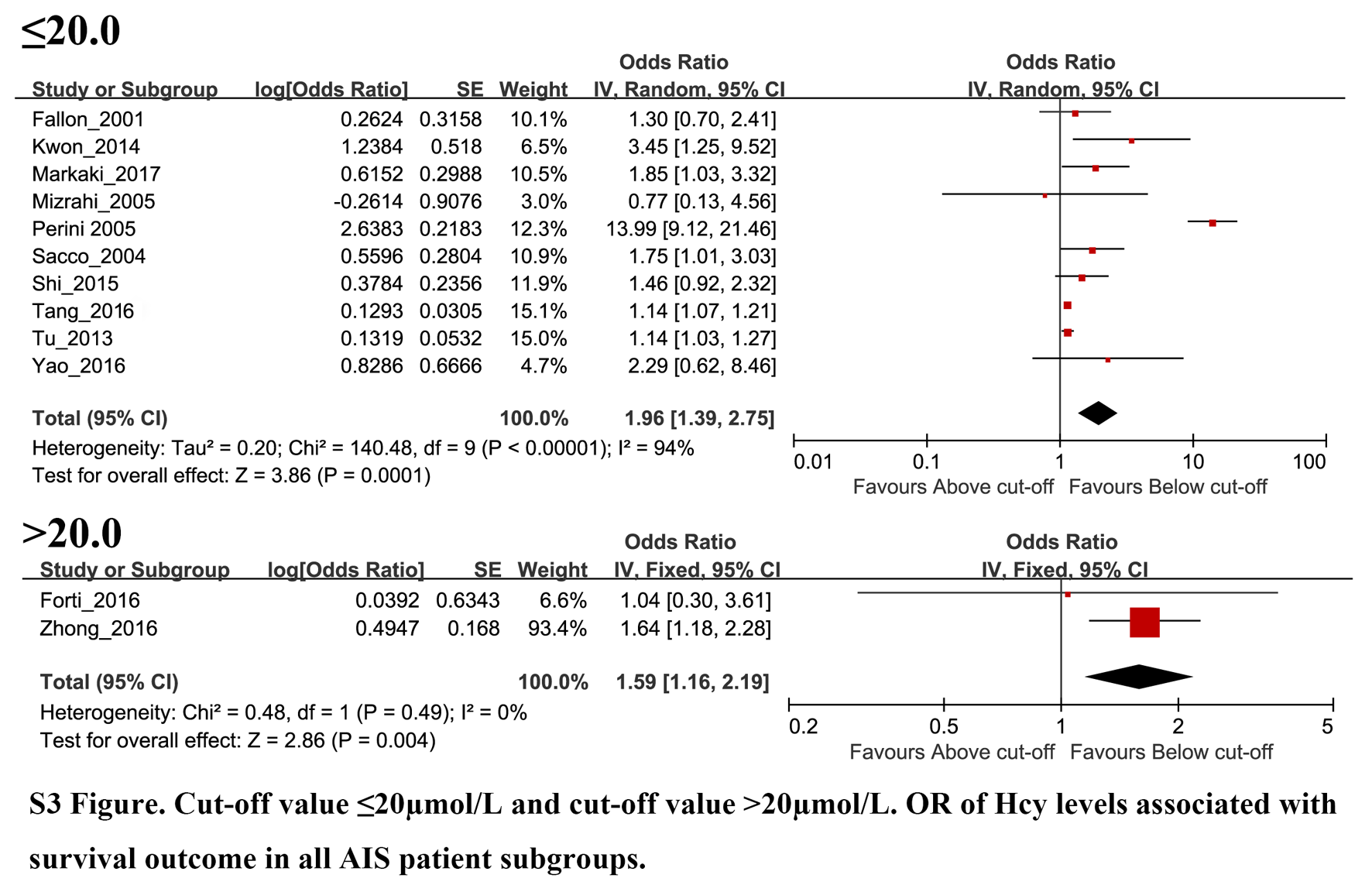

Supplement: Supplementary file 3 [file Image_3.tif]
