# Supplementary material for: The Prognostic Value of Homocysteine in Acute Ischemic Stroke Patients: A Systematic Review and Meta-Analysis
Source: Front Syst Neurosci. 2021 Feb 12;14:600582. doi: 10.3389/fnsys.2020.600582 (PMC7907516; doi:10.3389/fnsys.2020.600582)
Supplement: Supplementary file 4 [file Table_1.docx]

**S1 Table.** **OR values of outcome of AIS subgroups depended on cut-off value.**

| **Cutoff Value (μml/L)** | **Studies（n）** | **OR** | **95%CI** | **P value** | **Model** | **Heterogeneity** |
| --- | --- | --- | --- | --- | --- | --- |
|  |  |  |  |  |  | **Chi^2^, I^2^, P value** |
| **≤13.0** | 2 | 1.33 | 0.86-2.07 | 0.20 | Fixed | 0.38, 0%, 0.54 |
| **>13.0** | 9 | 1.71 | 1.36-2.17 | 0.00 | Random | 49.15, 84%, 0.00 |
| **≤16.5** | 7 | 1.29 | 1.13-1.49 | 0.0003 | Random | 13.00, 54%, 0.04 |
| **>16.5** | 3 | 2.62 | 0.74-9.33 | 0.14 | Random | 9.76, 80%, 0.008 |
| **≤20.0** | 10 | 1.96 | 1.39-2.75 | 0.0001 | Random | 140.48, 94%, 0.00 |
| **>20.0** | 2 | 1.59 | 1.16-2.19 | 0.004 | Fixed | 0.48, 0%, 0.49 |

OR: odds ratio; I^2^: I-squared; Fixed: Fixed, Inverse Variance model; Random: Random, I-V heterogeneity model.
